# Vitamin D deficiency in rheumatoid arthritis: prevalence, determinants and associations with disease activity and disability

**DOI:** 10.1186/ar3195

**Published:** 2010-11-29

**Authors:** Maurizio Rossini, Susanna Maddali Bongi, Giovanni La Montagna, Giovanni Minisola, Nazzarena Malavolta, Luigi Bernini, Enrico Cacace, Luigi Sinigaglia, Ombretta Di Munno, Silvano Adami

**Affiliations:** 1 Rheumatology Unit, University of Verona, Piazzale Stefani 1, 37124 Verona, Italy; 2 Rheumatology Unit, Università di Firenze, viale Pieraccini 18, 50139 Firenze, Italy; 3 Rheumatology Unit, II Università di Napoli, Via Pansini 5 80131 Napoli, Italy; 4 Rheumatology Unit, Ospedale San Camillo, Cir.ne Gianicolense 87-00152 Roma, Italy; 5 Rheumatology Unit, Università di Bologna, Via Massarenti 9, 40138 Bologna, Italy; 6 Rheumatology Unit, Università di Modena, Via del Pozzo 71, 41124 Modena, Italy; 7 Rheumatology Unit, Università di Cagliari, Via San Giorgio 12, 09100 Cagliari, Italy; 8 Rheumatology Unit, Istituto Ortopedico Gaetano Pini, Piazza Cardinal Ferraris 1, 20100 Milano, Italy; 9 Rheumatology Unit, Università di Pisa, Via Roma 67, 56126 Pisa, Italy

## Abstract

**Introduction:**

The aim of this study was to estimate the prevalence and determinants of vitamin D deficiency in patients with rheumatoid arthritis (RA) as compared to healthy controls and to analyze the association between 25-hydroxyvitamin D (25(OH)D) with disease activity and disability.

**Methods:**

The study includes 1,191 consecutive RA patients (85% women) and 1,019 controls, not on vitamin D supplements, from 22 Italian rheumatology centres. Together with parameters of disease activity, functional impairment, and mean sun exposure time, all patients had serum 25(OH)D measured in a centralized laboratory.

**Results:**

A total of 55% of RA patients were not taking vitamin D supplements; the proportion of these with vitamin D deficiency (25(OH)D level <20 ng/ml) was 52%. This proportion was similar to that observed in control subjects (58.7%). One third of supplemented patients were still vitamin D deficient. In non-supplemented RA patients 25(OH)D levels were negatively correlated with the Health Assessment Questionnaire Disability Index, Disease Activity Score (DAS28), and Mobility Activities of daily living score. Significantly lower 25(OH)D values were found in patients not in disease remission or responding poorly to treatment, and with the highest Steinbrocker functional state. Body mass index (BMI) and sun exposure time were good predictors of 25(OH)D values (*P *< 0.001). The association between disease activity or functional scores and 25(OH)D levels remained statistically significant even after adjusting 25(OH)D levels for both BMI and sun exposure time.

**Conclusions:**

In RA patients vitamin D deficiency is quite common, but similar to that found in control subjects; disease activity and disability scores are inversely related to 25(OH)D levels.

## Introduction

Vitamin D deficiency is extremely common in Europe and particularly in Southern countries where more than 90% of elderly people are affected [1-5]. Vitamin D deficiency is also frequent among young subjects: 25-hydroxyvitamin D (25(OH)D) levels lower than 20 ng/ml have recently been reported in Italy in almost a third of apparently healthy women [6].

Emerging evidence suggests that vitamin D plays an important role in immune regulation. Vitamin D receptors are found on several immune cells and *in vitro *studies have shown that vitamin D metabolites modulate T cell proliferation and dendritic cell function [7,8]. Epidemiological data also imply that vitamin D deficiency may be a risk for development of autoimmune and other chronic diseases [3,9].

Preliminary studies suggest that low levels of vitamin D may be common in rheumatoid arthritis (RA) [10-13]. More recently, vitamin D deficiency was found in 42 out of 145 postmenopausal women with RA in the USA, with the highest prevalence among African Americans [14,15]. Moreover, some authors reported an inverse relationship between serum levels of vitamin D metabolites and disease activity or disability in patients with RA or early inflammatory polyarthritis, although conflicting results have been found [12,15-17]

The aim of this study was to estimate the prevalence and determinants of vitamin D deficiency in patients with RA, and to analyze the association of vitamin D with disease activity and disability. We hypothesized that low levels of vitamin D would be common in patients with RA and inversely related to disease activity and disability.

## Materials and methods

### Patients and controls

The study population includes 1,191 consecutive patients (1,014 women, 177 men) from 22 rheumatology centres uniformly distributed across Italy (six northern, eight central, eight southern Italy).

The control group resulted from the merging of two population-based studies, representative of the general population recruited from osteoporosis centres equally distributed over the national territory of Italy. The first study included 700 Caucasian postmenopausal women aged 60 to 80 years not affected by diseases or on treatment expected to alter mineral metabolism [18]. The second study was made up of 608 premenopausal healthy women aged 20 to 50 years [6]. The characteristics of these two populations were described in details elsewhere and the data analyzed by the same coordinating centre. The aim of both studies was to investigate the prevalence, determinants and consequences of vitamin D deficiency in healthy Italian women [6,18].

### Clinical evaluation

All RA patients fulfilled the 1987 American College of Rheumatology (ACR) revised criteria for RA. The only inclusion criteria were a diagnosis of established RA and an age less than 75 years, irrespective of menopausal status. All patients were interviewed and examined at each clinical centre for the gathering of information on disease and treatment history.

Disease-related variables included disease onset and duration, presence of extra-articular manifestations, 28 tender joint count (TJC28) and 28 swollen joint count (SJC28). The three-variable Disease Activity Score (DAS 28) was calculated using C-reactive protein (CRP) and the Nijmegen formula: DAS28 = (0.56*sqrt(TJC28) + 0.28*sqrt(SJC28) + 0.36*ln(CRP+1)) * 1.10 + 1.15 [19]. The ACR criteria were considered in order to classify a patient as in remission at the time of observation. Clinical measures of disease related functional impairment included Health Assessment Questionnaire Disability Index (HAQ), Steinbrocker functional state and the mobility activities of daily living (ADL) [20-22]. RA specific treatment were collected and included the glucocorticoids, disease modifying antirheumatic drugs (DMARDs: methotrexate, cyclosporine, gold salts, sulfasalazine, antimalarials, and azathioprine) and the TNFα blockers (anti-TNF). Patients were interviewed regarding current use of drugs affecting bone metabolism including bisphosphonates, calcium and vitamin D supplements. Vitamin D supplements taken during the previous year were carefully evaluated and expressed as mean daily dose. Exposure to sunlight from March to September (sun exposure time) was quantified as <10, 10 to 20, 20 to 30 or >30 minutes daily. Body weight and height (Harpender stadiometer) were assessed and the body mass index (BMI = kg/m^2^) was calculated in all subjects.

### Laboratory assessment

Rheumatoid factor (RF), anti Cyclic Citrullinated Peptide (anti-CCP) and routine biochemistry were measured locally. Individual aliquots of serum samples were collected from June 2007 to May 2008 from each patient. Four aliquots were sent on dry ice by courier to the laboratory of the University of Verona, and kept at -70°C until the measurement of serum intact parathyroid hormone (PTH) and 25(OH)D using commercial ELISA kits (IDS Co., Bolden, UK) with inter-assay coefficient of variations ranging from 5 to 15%.

The study was in compliance with the Helsinki Declaration and was approved by the local Ethical Committees. The University Hospital of Messina was designated as the Coordinating Center, where the study protocol was approved on 2 February 2007. An informed written consent was obtained from all participants.

### Statistical analysis

All data management and analysis were centralized and conducted according to a prespecified plan by one of the centres. PTH and 25(OH)D were logarithmically transformed in order to normalize their distribution. The between subgroup differences were assessed by t-test or analysis of variance (ANOVA); analysis of covariance (ANCOVA) was used to adjust values for any confounding factor. Chi-square tests were used for categorical data.

Associations between continuous variables were examined using Pearson correlation coefficients and multivariate linear regression. Differences were considered significant at *P *< 0.05. All statistical procedures were carried out using a computer program (SPSS version 13.0, Inc., Chicago, IL, USA).

## Results

The RA sample was mostly female (85%) with a mean (± SD) age of 58.9 ± 11.1 years and disease duration of 11.5 ± 8.7 years. The main RA-related findings by gender are listed in Table 1, which includes also the main characteristics of the control women. The disease was somewhat more severe in women than in men. The volume of the serum sample from 23 patients was inadequate for the measurements with the auto-analyser.

**Table 1:** Main characteristics (means and confidence intervals or percentages) of study population by gender

	RA-women (*N *= 1,014)		RA-men (*N *= 177)		P_1_	Controls (*N *= 1,019)		P_2_
					
	Mean	95% C.I.	Mean	95% C.I.		Mean	95% C.I.	
Age (years)	58.7	58.0 to 59.4	60.0	58.4 to 61.6	n.s.	58.9	58.1 to 59.7	n.s.
BMI (kg/m^2^)	25.0	24.7 to 25.3	25.9	25.4 to 26.5	0.01	25.7	25.5 to 26.0	0.002
Disease duration (mo.)	138	131 to 145	138	119 to 157	n.s			
Swollen joint count (range 0 to 28)	3.12	2.81 to 3.43	1.98	1.53 to 2.43	0.003			
DAS 28	3.96	3.90 to 4.03	3.55	3.40 to 3.70	<0.001			
HAQ score (range 0 to 3)	1.18	1.13 to 1.24	0.87	0.75 to 0.99	<0.001			
ADL (range 4 to 16)	8.41	3.02 to 8.22	7.24	6.85 to 7.63	<0.001			
25(OH)D (ng/ml)	24.0	23.2 to 25.0	24.7	22.9 to 26.6	n.s.	19.3	18.4 to 20.2	<0.001
PTH (pg/ml)	25.0	24.1 to 25.9	24.9	23.2 to 26.6	n.s.	33.1	31.7 to 34.3	<0.001
Pre-menopause	35.7%					33.2%		ns
Sun exposure time >30 minutes	33.5%		51.4%		0.01	35%		ns
Smoking	20.8%		20.3%		n.s.	16.3%		<0.001
Extra-articular manifestations	15.3%		13.6%		n.s.			
Steinbrocker Functional state >1	66.8%		58.2%		n.s.			
DMARDs therapy	88.0%		89.3%		n.s.			
Anti to TNF therapy	46.5%		39.5%		n.s.			
ACR Remission	22.9%		24.9%		n.s.			
Good treatment response	43.7%		46.9%		n.s.			
Osteoporosis therapy	29.6%		13.6%		<0.01			
Glucocorticoid therapy	86.5%		83.1%		n.s.			
Rheumatoid Factor positive	63.3%		66.3%		n.s.			
Anti CCP positive	66.3%		66.7%		n.s.			

Abbreviations: 25(OH)D, 25-hydroxyvitamin D; ACR, American College of Rheumatology; ADL, activities of daily living; Anti-CCP, anti Cyclic Citrullinated Peptide; Anti-TNF, tumor necrosis factor α blocker; BMI, body mass index; CRP, C-reactive protein; DAS 28, Disease Activity Score 28; DMARDs, disease modifying antirheumatic drugs; HAQ, Health Assessment Questionnaire Disability Index; PTH, parathyroid hormone; RA, rheumatoid arthritis.

p_1 _is referred to between gender differences and p_2 _to between female patients and control women; n.s. = not significant.

Six hundred and thirty-four patients (55%) were not taking native vitamin D supplements; 16% and 27% of patients were taking ≤440 or ≥800 units of vitamin D_3 _per day, respectively.

The corresponding mean values of 25(OH)D and the proportion of patients with vitamin D deficiency with two different cut-off values are listed in Table 2.

**Table 2:** Mean and frequency of 25(OH)D values lower than 20 or 30 mg/ml according with ranges of vitamin D supplementations

D3 supplementation IU/day	N.	25(OH)D ng/ml mean (S.D.)	25(OH)D < 20 ng/ml (%)	25(OH)D < 30 ng/ml (%)
None	654	21.0 (10.2)	51.8%	84.4%
≤ 440	196	26.1 (12.4)	33.2%	74.5%
≥800	318	29.2 (18.5)	31.4%	63.5%
Controls	1,019	19.3 (14.3)^a^	65.0% ^a^	80.6% ^b^

^a ^= *P *< 0.01 and ^b ^= *P *< 0.05 versus supplemented RA patients.

Abbreviations: 25(OH)D (25-hydroxyvitamin D).

All differences (both proportions and means) across the three ranges of vitamin D supplementation in RA were statistically significant.

In patients not taking vitamin D supplements, a significant negative correlation between 25(OH)D serum levels and age was observed (*P *< 0.05), and mean values from June to December were significantly higher than from January to May (23.5 versus 19.0 ng/ml, respectively, data not shown).

Among patients not vitamin D supplemented the logarithm of the 25(OH)D levels (Ln 25(OH)D) levels were significantly correlated with logarithm of PTH, BMI, DAS28, HAQ score and ADL (Table 3). The age and BMI adjusted mean 25(OH)D levels were not statistically different in non-supplemented RA patients (19.7 ± 10.0 ng/ml) and control women (19.2 ± 9.9 ng/ml). Table 4 shows 25(OH)D levels by categorical variables in patients not vitamin D supplemented.

**Table 3:** Correlations between the natural logarithm of 25(OH)D (Ln 25(OH)D) and several continuous variables in patients not taking vitamin D supplements

	Correlation coefficients (95% C.I.)	Regression Coefficients (r)	*P*
Ln PTH pg/ml	-0.076 (-0.121, -0.031)	0.114	0.031
BMI (kg/m^2^)	-0.015 (-0.022 , -0-007)	0.135	<0.001
Swollen joints count	-0.009 (-0.017, -0.002)	0.087	0.121
DAS28	-0.091 (-0.129, -0.052	0.191	<0.001
HAQ score	-0.101 (-0.140, -0.062)	0.175	<0.001
ADL	-0.027 (-0.038, -0.016	0.165	<0.001

Abbreviations: 25(OH)D, 25-hydroxyvitamin D; ADL, activities of daily living; BMI, body mass index; DAS 28, Disease Activity Score 28; HAQ, Health Assessment Questionnaire Disability Index; Ln PTH, natural logarithm of parathyroid hormone.

**Table 4:** Mean 25(OH)D levels and proportion of patients with 25(OH)D levels <20 ng/ml according to categorical variables in non vitamin D supplemented patients

Categorial variable		Mean	Standard deviation	*P*	% with 25(OH)D <20 ng/ml	*P*
Disease Remission	No	21.7	11.3	0.008	51.6%	<0.001
	Yes	24.2	12.1		35.4%	

DAS28	<3.1	23.1	9.9	0.008	34.1%	<0.001
	3.1 to 5.1	20.2	11.8		59.3%	
	>5.1	19.4	9.2		62.0%	

Steinbrocker Functional state	1	24.3	13.0	<0.001	40.7%	0.001
	>1	21.1	10.4		52.0%	

Treatment Response	Good	23.6	13.1		41.4%	<0.001
	Fair	21.5	10.0	0.010	51.2%	
	No	20.4	10.4		58.8%	

Sun exposure time	<10 minutes	20.7	10.3	0.008	50.5%	0.049
	10 to 20 minutes	21.1	9.1		52.3%	
	20 to 30 minutes	22.3	12.1		51.4%	
	>30 minutes	23.9	13.0		42.5%	

Abbreviations: 25(OH)D, 25-hydroxyvitamin D; ANOVA, analysis of variance); DAS 28, Disease Activity Score 28. *P-*values were obtained by ANOVA and Chi square test.

Significantly lower 25(OH)D values were found in patients not experiencing disease remission or with DAS28 >5.1 or poorly responding to treatment, and with the highest Steinbrocker functional state. Time spent outdoors during summer months (sun exposure score) was a good predictor of 25(OH)D levels.

Patients with the worse indices of disease activity or disability were spending significantly less time in sunshine (Table 5) and were then more likely to develop a vitamin D deficiency.

**Table 5:** Association between daily sun exposure time (minutes) and parameters of disease activity or disability (percent of patients or mean ± SD)

Sun exposure time (minutes)	<10	10 to 20	20 to 30	>30	*P *= Chi-square or ANOVA
Disease Remission = No	85.8%	70.3%	76.3%	73.7%	0.007
Treatment Response = Fair or No	61.1%	54.6%	50.6%	52.9%	0.05
Steinbrocker Functional state > 1	69.6%	66.5%	59.0%	57.4%	0.001
Swollen joints count	4.3 ± 6.3	3.2 ± 4.9	2.4 ± 4.0	2.1 ± 3.3	<0.001
DAS28 >3.1	83.1%	73.7%	70.9%	65.5%	<0.001
HAQ score	1.4 ± 0.9	1.2 ± 0.9	1.0 ± 0.8	0.9 ± 0.7	<0.001

Abbreviations: DAS 28, Disease Activity Score 28; HAQ, Health Assessment Questionnaire Disability Index.

Thus, the association between disease activity or disability and 25(OH)D levels was re-examined for values adjusted for both sun exposure time and BMI, another strong determinant of 25(OH)D values. The results of this re-analysis are listed in Table 6.

**Table 6:** Mean 25(OH)D levels adjusted for BMI and sun exposure time according to categorical variables in non vitamin D supplemented patients

Categorial variable		Mean	Standard deviation	*P*
Disease remission	No	21.8	11.1	0.057
	Yes	23.6	12.1	

Steinbrocker functional state	1	24.0	12.8	0.001
	>1	21.2	10.1	

DAS28	<3.1	22.6	9.9	<0.001
	>3.1	20.3	10.7	

Treatment response	Good	23.4	12.1	0.020
	Fair	21.5	9.9	
	No	20.5	10.1	

Abbreviations: ANOVA, analysis of variance; 25(OH)D, 25-hydroxyvitamin D; BMI, body mass index; DAS 28, Disease Activity Score 28. *P-*values were obtained by ANOVA and Chi square test.

Even after these adjustments, most indices of disease activity or disability remained significantly related with 25(OH)D levels and the correlations between Ln 25(OH)D levels and HAQ score or ADL remained significant (*P *= 0.002 and 0.001, respectively; results not shown).

## Discussion

We have found that vitamin D deficiency (25(OH)D values <20 ng/ml) is common in RA patients affecting 43% of the entire cohort. Our results are similar to those reported by others in smaller sample sizes, with a prevalence of vitamin D deficiency ranging from 30 to 63% [10-15].

In our study the proportion of patients with vitamin D deficiency rises to 52% in patients not taking vitamin D supplements, but it was also unacceptably high in women on approximately 400 or 800 U vitamin D daily supplements, that is, 33 and 31% respectively. These latter proportions rise considerably if the threshold 25(OH)D levels are set to 30 ng/ml, but they are very similar to those we observed in control women representative of the general population and this indicates that vitamin D deficiency is, at least in Italy, a general problem.

From a careful analysis of a large number of epidemiological studies it was recently found that the optimal 25(OH)D concentrations for bone health and extra-skeletal benefits are between 36 to 40 ng/ml% [23]. These levels were achieved only by 9% of our patients (results not shown) and this indicates that, at least in RA patients, in order to achieve 25(OH)D levels above 38 ng/ml in more than 90% of the population, the daily dose of vitamin D should be substantially higher than 800 U per day.

As expected, the logarithmic values of 25(OH)D were negatively (*P *< 0.04) correlated with the logarithmic levels of PTH, but only in patients not on vitamin D supplements.

In non-supplemented patients, 25(OH)D levels were associated with several variables. Sun exposure and BMI are well established risk factors for vitamin D deficiency and these associations are confirmed in the present study [24].

The scope of this study was to evaluate to what extent vitamin D deficiency was related in RA patients with the severity of the disease. The inverse relationships between vitamin D levels and disease activity or functional impairment are of interest but not of obvious interpretation. Similar relationships have been found also by others. Cutolo *et al. *reported a significant inverse association between 25(OH)D and DAS28 in patients with active RA and Patel *et al. *found an inverse relationship between 25(OH)D levels and tender joint count, DAS28, and HAQ score only at disease onset, but not in patients with a disease duration longer than one to two years [16,17]. Though our study population included a large variety of disease activity, treatments and disease durations, the correlations we found were all highly significant and this is likely explained by the size of our study, 6- to 10-fold larger than previous studies. We found that the worse the direct or indirect indices of disease activity, the lower the 25(OH)D levels or the higher the proportion of patients with vitamin D deficiency. Thus, the proportion of patients with vitamin D deficiency was around 30% lower in patients on disease remission or judged as good responders to treatment or for a DAS28 <3.1. In addition, both functional indices and mobility ADL were negatively associated with 25(OH)D levels with a *P *< 0.001.

At a first glance, the most obvious explanation for these findings is that patients with very active disease are at higher risk of vitamin D deficiency rather than the other way around. Indeed, we found an association between sun exposure time and achievement of disease remission, good treatment response, Steinbrocker's functional state, HAQ and swollen joint counts. This indicates that patients with uncontrolled RA and/or with severe functional impairment are less prone to spend time outdoors in sunshine and are, therefore, at higher risk of vitamin D deficiency. Thus, the conclusions drawn in previous cross-sectional studies regarding the immunomodulatory role played by vitamin D in inflammatory arthritis, should be interpreted with caution, if 25(OH)D values are not adjusted for the known risk factors for vitamin D deficiency [16,17]. However, when the correlations between disease activity scores and vitamin D deficiency were reanalysed by adjusting the 25(OH)D levels for sun exposure and BMI, the association remained statistically significant for Steinbrocker's functional state, DAS28, treatment response, HAQ score and mobility ADL. These results indicate that patients with very active RA are at higher risk of vitamin D deficiency for similar BMI and sun exposure, for reasons that remain unknown.

The main strength of this study is its size. There are also important limitations. The large heterogeneity in terms of specific treatments and disease duration hampers the interpretation of some associations but it is of help for defining the risk of developing vitamin D deficiency. Our control group was identified *a posteriori *and it was not perfectly matched since it does not include men and women aged 50 to 60 years.

## Conclusions

In this study we found that in RA, patients' vitamin D deficiency is quite common, but not more common than in age-matched control women representative of the general population. 25(OH)D levels were strongly inverse related to disease activity and disability scores. The causality of these associations remain to be assessed in longitudinal studies aimed at evaluating the clinical response to a vitamin D supplementation dose regimen large enough to increase 25(OH)D levels over 38 ng/ml [25].

## Abbreviations

25(OH)D: 25-hydroxyvitamin D; ACR: American College of Rheumatology; ADL: activities of daily living; ANCOVA: analysis of covariance; ANOVA: analysis of variance; Anti-CCP: anti Cyclic Citrullinated Peptide; Anti-TNF: tumor necrosis factor α blocker; BMI: body mass index; CRP: C-reactive protein; DAS 28: Disease Activity Score 28; DMARDs: disease modifying antirheumatic drugs; HAQ: Health Assessment Questionnaire Disability Index; PTH: parathyroid hormone; RA: rheumatoid arthritis; RF: rheumatoid factor; SJC28: 28 swollen joint count; TJC28: 28 tender joint count.

## Competing interests

The authors declare that they have no competing interests.

## Authors' contributions

SA, GM, LS, OD, MR and FG conceived of the study, and participated in its design and coordination and helped to draft the manuscript. SA drafted the manuscript and performed the statistical analysis. All authors provided a large proportion of the study population, and read and approved the final manuscript.
